# Enhanced As(III) adsorption-oxidation via synergistic interactions between bacteria and goethite

**DOI:** 10.1016/j.eehl.2024.12.001

**Published:** 2024-12-18

**Authors:** Jie Deng, Shaowei Mi, Chenchen Qu, Qiaoyun Huang, Xionghan Feng, Xiaoming Wang

**Affiliations:** Key Laboratory of Arable Land Conservation (Middle and Lower Reaches of Yangtze River), Ministry of Agriculture and Rural Affairs, State Environmental Protection Key Laboratory of Soil Health and Green Remediation, Hubei Key Laboratory of Soil Environment and Pollution Remediation, College of Resources and Environment, Huazhong Agricultural University, Wuhan 430070, China

**Keywords:** As(III)-Oxidizing bacteria, Fe (oxyhydr)oxides, Arsenic, Adsorption-oxidation, Humic acid

## Abstract

The adsorption and oxidation of arsenite [As(III)] by soil components are critical processes that influence its toxicity and mobility. However, the specific mechanisms driving the synergistic interactions among bacteria, soil minerals, and humic acid (HA) in these processes remain insufficiently understood. This study investigated the effects of goethite and HA association on As(III) adsorption-oxidation by the As(III)-oxidizing bacterium SY8 using batch incubation experiments and spectroscopic analyses. The results indicated that goethite inhibited the growth of SY8, but its binary and ternary composites with HA and SY8 substantially enhanced the adsorption and oxidation of As(III) compared to SY8 alone. This enhancement could be attributed to the generation of hydroxyl radicals (·OH) through Fenton-like reactions that contribute to the enhanced oxidation of As(III). The Fenton-like reactions involved interactions between H_2_O_2_ and goethite, as well as the activation of molecular O_2_ by structural Fe(II). Furthermore, the proportion of As(V) associated with the solids was lower than that in the solution, suggesting that As(III) oxidation by SY8 was potentially inhibited by As(III) adsorption on goethite. Additionally, HA did not affect SY8 growth or its As(III) oxidation capability, but slightly enhanced As adsorption on the composites. These findings reveal a complex interplay among microbial, mineral, and organic matter interactions. Understanding these interactions is essential for elucidating soil As biogeochemical processes and developing effective remediation strategies for As-contaminated environments.

## Highlights

• Effects of goethite and HA on As(III) adsorption and oxidation by bacteria were evaluated.
• Goethite substantially enhanced both the adsorption and oxidation of As(III) by the bacteria.
• ·OH generation contributed to the enhanced oxidation of As(III) on the goethite-bacteria composites.
• As(III) adsorption on goethite surfaces inhibited its subsequent oxidation by the bacteria.
• HA did not alter bacterial growth and its ability to oxidize As(III) but slightly enhanced As adsorption.

## Graphical abstract

**Figure:**
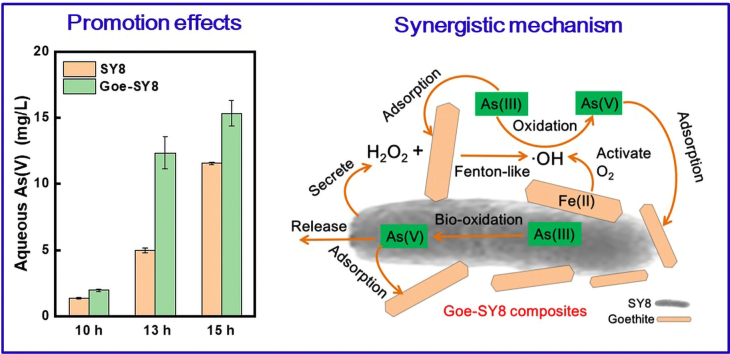

## 1 Introduction

Arsenic (As), a toxic metalloid widely distributed in soils globally, poses substantial ecological and human health risks due to its complex geochemical behavior [1,2]. The predominant inorganic forms of As in soils, arsenite [As(III)] and arsenate [As(V)], are strongly influenced by redox conditions. The As(III) is notably more toxic, mobile, and soluble than As(V), thereby posing a greater threat to ecosystems and human health [3,4]. Therefore, the oxidation of As(III) to the less toxic As(V), facilitated by mineral surface reactions, microbial activities, and interactions with organic matter, represents a critical strategy for As remediation, as it reduces both the toxicity and mobility of As [5]. The adsorption and oxidation processes of As(III) involve complex interfacial reactions among minerals, microorganisms, and organic matter in soils, highlighting the need for an interdisciplinary approach to fully elucidate the speciation and environmental fate of As [6]. A comprehensive understanding of As(III) behavior across these multi-interfaces within soil components is essential for advancing effective remediation strategies.

Microorganisms play a pivotal role in regulating the biogeochemical cycles of As in soils, significantly influencing both As speciation and mobility [7]. These organisms interact with As through various processes, including sorption to cell surfaces, intracellular accumulation, and complex metabolic pathways [8]. To adapt to As toxicity, microorganisms have evolved sophisticated mechanisms, such as As(III) oxidation to the less toxic As(V) and As methylation for detoxification [9,10]. Research has identified numerous As-resistant bacteria in contaminated soils, particularly those capable of As(III) oxidation and As(V) reduction [11,12]. Key bacterial mechanisms for As(III) metabolism include transport of As(III) into the periplasmic space, where it is oxidized to As(V) by As oxidase [8]. In addition to As-resistant bacteria, soil minerals, particularly Fe (oxyhydr)oxides and humic substances (HS), also play significant roles in As mobility and transformation [13]. Fe (oxyhydr)oxides can adsorb As through inner–sphere complexes and may facilitate As(III) oxidation via interfacial catalytic effects [14,15]. HS are complex organic compounds in soils, mainly composed of humic acid (HA), fulvic acid, and humin, which originate from the breakdown and polymerization of biogenic materials like plant cuticles, cellulose, tannins, and lignin. Their intricate molecular structures, including aromatic rings, carboxyl, and phenolic hydroxyls, confer high chemical stability and microbial resistance, making them persistent in soils [16,17]. The HS can complex with As and compete with mineral surfaces for adsorption sites, thereby influencing As mobility and bioavailability in soils [[18], [19], [20]].

In soil environments, the interactions among bacteria, minerals, and organic matter components alter the physico-chemical and biological properties in complex ways, beyond what can be observed when these components exist independently of each other [21]. Specifically, interactions between Fe (oxyhydr)oxides and bacteria play a central role in influencing the extent of As adsorption and redox reactions at microbial-mineral interfaces, thus affecting the mobility and toxicity of As. As-resistant bacteria may compete with As species for adsorption sites on Fe (oxyhydr)oxides, potentially altering As mobility and heightening environmental risks [[22], [23], [24]]. Adsorption of As on Fe (oxyhydr)oxides significantly inhibits the reduction of As(V) and the oxidation of As(III), mediated by redox-active bacteria, thereby impacting As speciation [13,23,25]. As(III)-oxidizing bacteria inherently associate with Fe (oxyhydr)oxides, forming natural composites. However, the specific mechanisms driving As(III) adsorption and oxidation within these binary composites remain to be fully elucidated. In addition, naturally occurring Fe (oxyhydr)oxides often interact with HS via adsorption and co-precipitation, with these associations markedly influencing As adsorption dynamics [26,27]. Given their potential impact on As mobilization and transformation, the complex interfacial reactions within ternary composites of Fe (oxyhydr)oxides, As(III)-oxidizing bacteria, and HA require further comprehensive investigation.

This study aims to elucidate the adsorption-oxidation processes and underlying mechanisms of As(III) at the multi-interfaces of Fe (oxyhydr)oxides, bacteria, and HA within binary and ternary composite systems. Goethite (Goe) was chosen as the representative Fe (oxyhydr)oxides due to its stable and well-defined crystal structure, constant chemical composition, high surface reactivity towards As, and widespread distribution in various soil environments [28]. The As(III)-oxidizing bacterium SY8, goethite, HA, and their composites were prepared and characterized using X-ray diffraction (XRD), scanning electron microscopy (SEM), Fourier-transform infrared spectroscopy (FTIR), and zeta potential (ζ) measurements. The growth curve of SY8 was evaluated at different pH values and concentrations of As(III), HA, and goethite, while the As(III) oxidation kinetics of SY8 was assessed at various pH and As(III) concentrations. Besides, the adsorption-oxidation processes and mechanisms of As(III) at the interfaces of Goe-SY8-HA composites were investigated by batch incubation experiments in combination with As K-edge X-ray absorption near-edge structure (XANES) spectroscopy, X-ray photoelectron spectroscopy (XPS), acidic dissolution assays, and hydroxyl radical (·OH) quantification. These insights are crucial for a comprehensive understanding of the environmental biogeochemical dynamics of As, facilitating advancements in As remediation technologies.

## 2 Materials and methods

### 2.1 Preparation and characterization of goethite, HA, SY8, and their composites

The heterotrophic As(III)-oxidizing bacterium SY8, isolated from As-contaminated soil [29,30], was revived from a frozen glycerol tube and cultured to the log phase in Luria–Bertani (LB) medium. The taxonomic analysis, presented in Fig. S1, identifies SY8 as belonging to the genus *Achromobacter* within the phylum β-Proteobacteria. Following cultivation, the cells were centrifuged, rinsed with sterile deionized (DI) water, and stored at 4 °C for use within 24 h. The bacterial suspension was adjusted to a cell mass concentration of 10 g/L prior to experiments. The HA (Sigma Aldrich) was purified, according to Qu et al. [31], by dissolving HA in sterile DI water at alkaline pH, followed by stirring, centrifugation, adjustment of the HA concentration to 1 g/L and pH 7, and storage in darkness. The goethite was synthesized following the procedures described by Weng et al. [32]. The synthesized goethite was autoclaved to ensure sterility for biological experiments [13] and diluted to 20 g/L with sterile DI water.

The binary (Goe-SY8 and HA-SY8) and ternary (Goe-HA-SY8) composites were prepared by mixing equal volumes (10 mL each) of 10 g/L SY8, 20 g/L goethite, and 1 g/L HA suspensions, followed by stirring for 2 h, resulting in a total volume of 20 mL for binary composites and 30 mL for ternary composites. For the As(III) adsorption-oxidation experiments, 10 mL of binary composite suspension and 15 mL of the ternary composites suspension were respectively added to 90 mL and 85 mL of an As(III) and LB mixed solution, resulting in final concentrations of 0.5 g/L SY8, 1 g/L goethite, and 50 mg/L HA in the systems. The structural, morphological, and surface properties of the composites were characterized using XRD, SEM, FTIR, and ζ measurements, with detailed methods provided in the supporting information (Text SI-1).

### 2.2 The growth curve of SY8 and As(III) oxidation kinetics

The As(III) stock solution was prepared by dissolving As_2_O_3_ in 2 M NaOH, diluted with sterile DI water to a concentration of 200 mg/L, and adjusted to pH 7. The effects of initial pH (4–10), As(III) concentration (20–200 mg/L), HA concentration (0–100 mg/L), and goethite concentration (0–2 g/L) on the growth of SY8 were determined using an automated microbial growth curve detection system (FLUOstar OMEGA, BioDot, USA). The concentrations of As and HA used in this study are representative of levels that might be found in the soil solution of highly As-contaminated soil [33,34]. The optical density (OD_600_) of the SY8 suspension was monitored every 1 h for 24 h, with the growth curve derived by subtracting the background OD_600_ value at 0 h. The modified Gompertz model [35], a standard S-shaped growth curve model, was employed to analyze the growth responses of SY8 to varying conditions. This model allowed for the estimation of the maximum OD_600_ value (Y_max_) and lag phase (λ) as follows:

$$Y_{t}=Y_{0}+\left(Y_{\max}\;–\;Y_{0}\right)\times \exp\left\{–\exp\left[–\mu_{\max}\times \left(t\;–\;t_{\max}\right)\right]\right\}$$

$$\lambda =t_{\max}\;–\;1/\mu_{\max}$$

where Y_t_ represents the value of OD_600_ at incubation time t, indicating the microbial density; Y_max_ and Y_0_ denote the maximum and minimum OD_600_ values, respectively; μ_max_ (h^−1^) is the growth rate constant, indicating the maximum value of the growth rate; t_max_ (h) is the time at μ_max_; and λ (h) is the lag phase.

The As(III) oxidation kinetics by 0.5 g/L SY8 were assessed at initial pH values of 5.5, 7, 8.5, and 10 and at As(III) concentrations of 20, 50, 100, and 200 mg/L, reflecting conditions commonly found in As-contaminated environments [36]. These experiments were conducted at 30 °C with an agitation rate of 180 r/min in an LB culture medium to simulate optimal growth conditions for SY8. To accurately monitor the oxidation process, 3 mL of the culture suspension was periodically withdrawn at predetermined intervals and immediately filtered through a sterile 0.22 μm membrane to separate soluble As(III) and As(V) from the biomass. The As(III) oxidation kinetics at each condition were conducted in triplicate to ensure reproducibility and reliability. The concentrations of soluble As(III) and As(V) in the filtered samples were quantitatively determined using atomic fluorescence spectrometry (AFS, Kylin S12, Jitian) (Text SI-2).

### 2.3 As(III) adsorption-oxidation kinetics on Goe-HA-SY8 binary and ternary composites

To assess the effects of goethite and HA association on As(III) adsorption and oxidation by SY8, batch kinetics experiments were conducted using 20 mg/L As(III) at pH 7, 30 °C, and 180 r/min. The experiments included the individual components (SY8 and goethite), binary composites (Goe-SY8 and HA-SY8), and ternary composites (Goe-HA-SY8). All reactions were operated in the dark to prevent photocatalytic oxidation of As(III) [37,38]. For each setup, the As(III) stock solution was initially mixed with LB culture medium. Subsequently, solid suspensions were added to the mixed solution to achieve a final volume of 100 mL with 0.5 g/L SY8, 1 g/L goethite, and 50 mg/L HA. Each condition was conducted in triplicate. At predetermined intervals, the suspension pH was measured, and 3 mL of the sample was withdrawn for immediate filtration through a sterile 0.22 μm membrane to quantify soluble As(III) and As(V) concentrations using AFS. Additionally, for selected time points, a 10 mL sample was centrifuged, freeze-dried, and analyzed using As K-edge XANES spectroscopy and XPS to determine the speciation and oxidation state changes of As, O, and Fe.

To extract acidic-soluble Fe from the suspension, a 3 mL sample was mixed with an equal volume of 1 M HCl, stirred for 24 h, and then filtered through a sterile 0.22 μm membrane. The concentration of soluble Fe in the filtered samples was determined using an inductively coupled plasma optical emission spectrometer (ICP-OES, Agilent). To quantify the generated ·OH, terephthalic acid (TPA, 10 μM) was added at the beginning of the experiments in the goethite, Goe-SY8, and Goe-HA-SY8. After 33 h, a 5 mL suspension sample was withdrawn, centrifuged, and filtered. The concentration of ·OH in the filtrate was measured using high-performance liquid chromatography (HPLC, Agilent 1260) equipped with a fluorescence detector [39] (Text SI-2).

### 2.4 As K-edge XANES spectroscopy and XPS analyses

As K-edge XANES spectroscopy was employed to determine As speciation in Goe-SY8 and Goe-HA-SY8 samples after reaction for 4, 13, 15, and 33 h. These time points were selected based on preliminary kinetic data indicating critical stages in As(III) oxidation. The spectra were collected at BL11B of the Shanghai Synchrotron Radiation Facility (SSRF) using a double crystal monochromator Si(111) in fluorescence mode. To optimize the signal-to-noise ratio, approximately 20 mg of each sample was pressed into discs and affixed to tape. Two scans were collected for each sample. The acquired spectra were averaged and processed, including background removal, normalization, and linear combination fitting (LCF) analyses using the Athena program [40]. The LCF analysis was conducted within the energy range of 11,856–11,906 eV using adsorbed As(III) and As(V) as reference standards (Fig. S2). The XPS was conducted to analyze the surface chemistry of the solid products, focusing on changes in the Fe 2p and O 1s regions. Full and narrow spectra were collected at 0, 13, and 23 h to capture the variations in surface interactions using a Thermo Scientific K-Alpha spectrometer. The detailed acquisition parameters and data analysis procedures are provided in the supporting information (Text SI-1).

## 3 Results and discussion

### 3.1 Growth curve of SY8 at different conditions

**Fig. 1:**
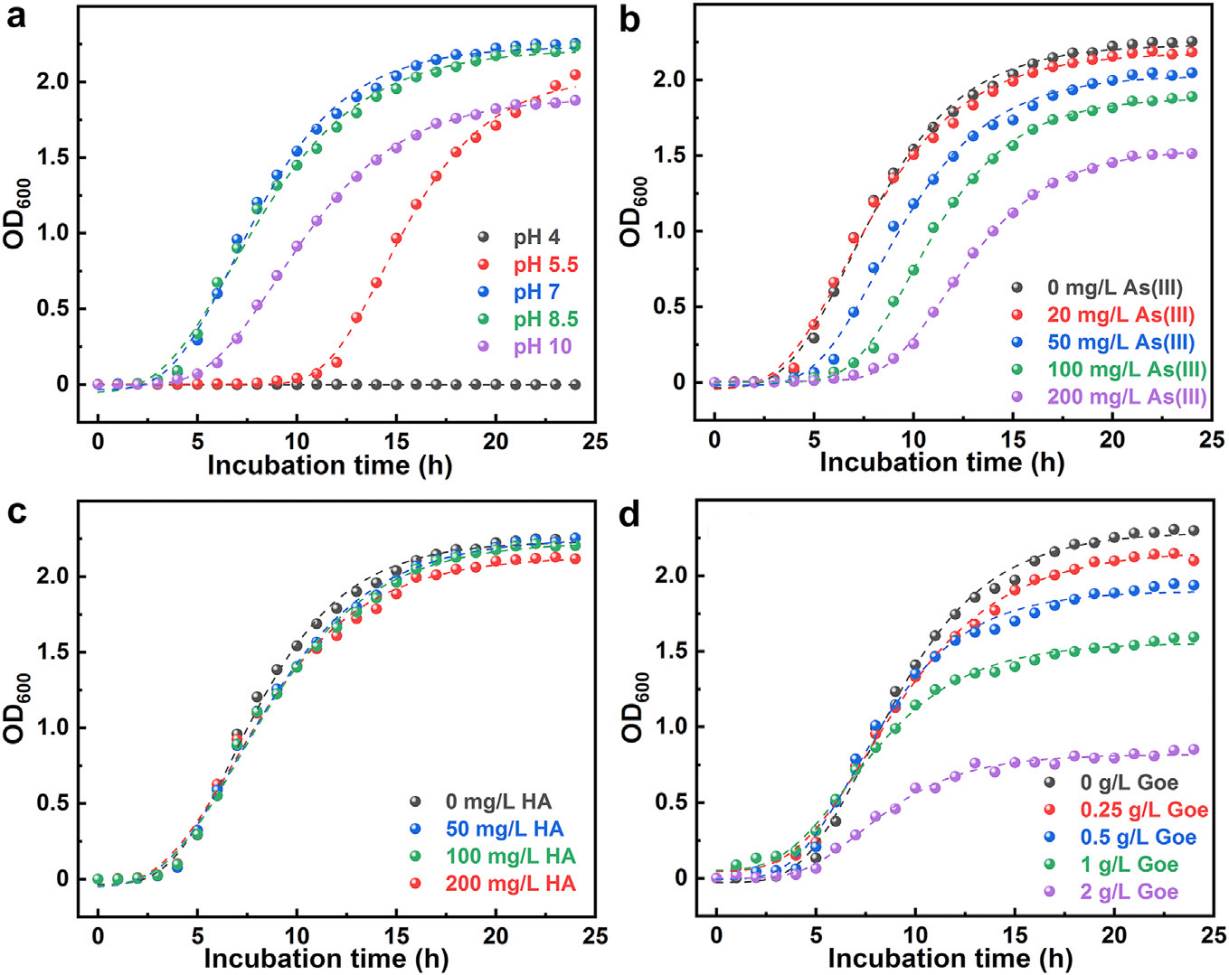
Effects of pH (a), As(III) concentration (b), and HA (c) and goethite coexistence (d) on the growth of SY8. The dashed line is the modified Gompertz model fits, the relevant parameters are summarized in Table S1.

At initial pH values of 7 and 8.5, the growth curve of SY8 showed a typical bacterial growth profile with three distinct phases: lag, log (exponential), and stationary. The log phase, marked by rapid cell division, occurred approximately at 4–17 h, transitioning into the stationary phase around 17–24 h (Fig. 1). The growth of SY8 was significantly inhibited at pH 4 (Fig. 1a), probably attributed to protein denaturation and enzyme inactivation under strongly acidic conditions [41]. The Gompertz model fitting parameter λ was notably higher at initial pH 10 and 5.5 compared to pH 7 and 8.5 (Fig. 1a and Table S1), indicating a delayed onset of the log phase, particularly pronounced at pH 5.5. As the initial As(III) concentration increased, the Y_max_ and λ values of the growth curves showed a decreasing and increasing trend, respectively (Fig. 1b and Table S1), which indicates that elevated As(III) concentrations exerted a toxic effect on the SY8 growth, significantly reducing maximum biomass and delaying the log phase at higher As(III) loadings. The concentration of HA had minimal impact on the growth of SY8 (Fig. 1c and Table S1), suggesting that SY8 primarily utilized the readily available organic matter from the LB medium rather than HA as a carbon source, as HA is recalcitrant and not easily decomposed [42]. In contrast, goethite exhibited an inhibitory effect on SY8 growth, and the inhibitory effect intensified with increasing mineral concentration, although it did not significantly delay the log phase (Fig. 1d and Table S1). This interaction highlights the potential influence of SY8-mineral dynamics on As fate and transport in the environment.

### 3.2 As(III) oxidation kinetics by SY8 at different As concentrations and pH values

**Fig. 2:**
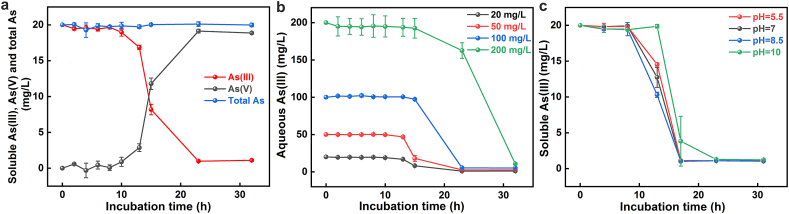
The oxidation process of As(III) by SY8 at pH 7 and 20 mg/L As(III) (a) and the effects of initial As(III) concentration (b) and solution pH (c) on As(III) oxidation by SY8 with incubation time, corresponding data of soluble As(V) are given in Fig. S3a and b.

At an initial As(III) concentration of 20 mg/L and pH 7, SY8 fully oxidized soluble As(III) to As(V) within 23 h (Fig. 2a), suggesting the bacterium's potential for As bioremediation. During the lag phase, which lasted approximately 8 h, minimal As(III) oxidation occurred (Fig. 2a), correlating with SY8's slow growth rate during this period (Fig. 1b). In the subsequent log phase from 8 to 17 h, the oxidation of As(III) by SY8 was significantly accelerated, resulting in complete conversion to As(V) within 23 h (Fig. 2a). This phase demonstrated a direct relationship between SY8's growth and its enhanced As(III) oxidation capability. The total soluble As concentration remained nearly constant throughout the incubation (Fig. 2a), which suggests that minimal As was sorbed (e.g., adsorption, intracellular storage) by SY8, and the intracellular As(III) oxidation likely served solely as a detoxification process [43].

When the initial As(III) concentration increased to 50 mg/L and 100 mg/L at pH 7, SY8 was still able to completely oxidize As(III) to As(V) within 23 h (Figs. 2b and S3a). However, at an initial As(III) concentration of 200 mg/L, a noticeable toxic effect on SY8 was observed, reflected in inhibited and delayed oxidation activity (Fig. 1, Fig. 2b). This suggests a critical threshold at which the toxicity of As exceeds SY8's detoxification capacity, thereby impacting the growth of SY8 and As(III) oxidation efficiency. The rate of As(III) oxidation by SY8 was notably influenced by solution pH, with optimal activity observed under neutral to slightly alkaline conditions, following the order pH 8.5 > pH 7 > pH 5.5 > pH 10 (Figs. 2c and S3b). This pH dependency, which aligns with SY8's growth patterns (Fig. 1a), emphasizes the important role of environmental pH in modulating microbial proliferation and As detoxification. SY8's optimal As oxidation performance under neutral to slightly alkaline conditions, with reduced efficacy at extreme pH values, highlights the necessity for pH management in As bioremediation strategies.

### 3.3 As(III) adsorption-oxidation kinetics on goethite, HA and SY8 composites

**Fig. 3:**
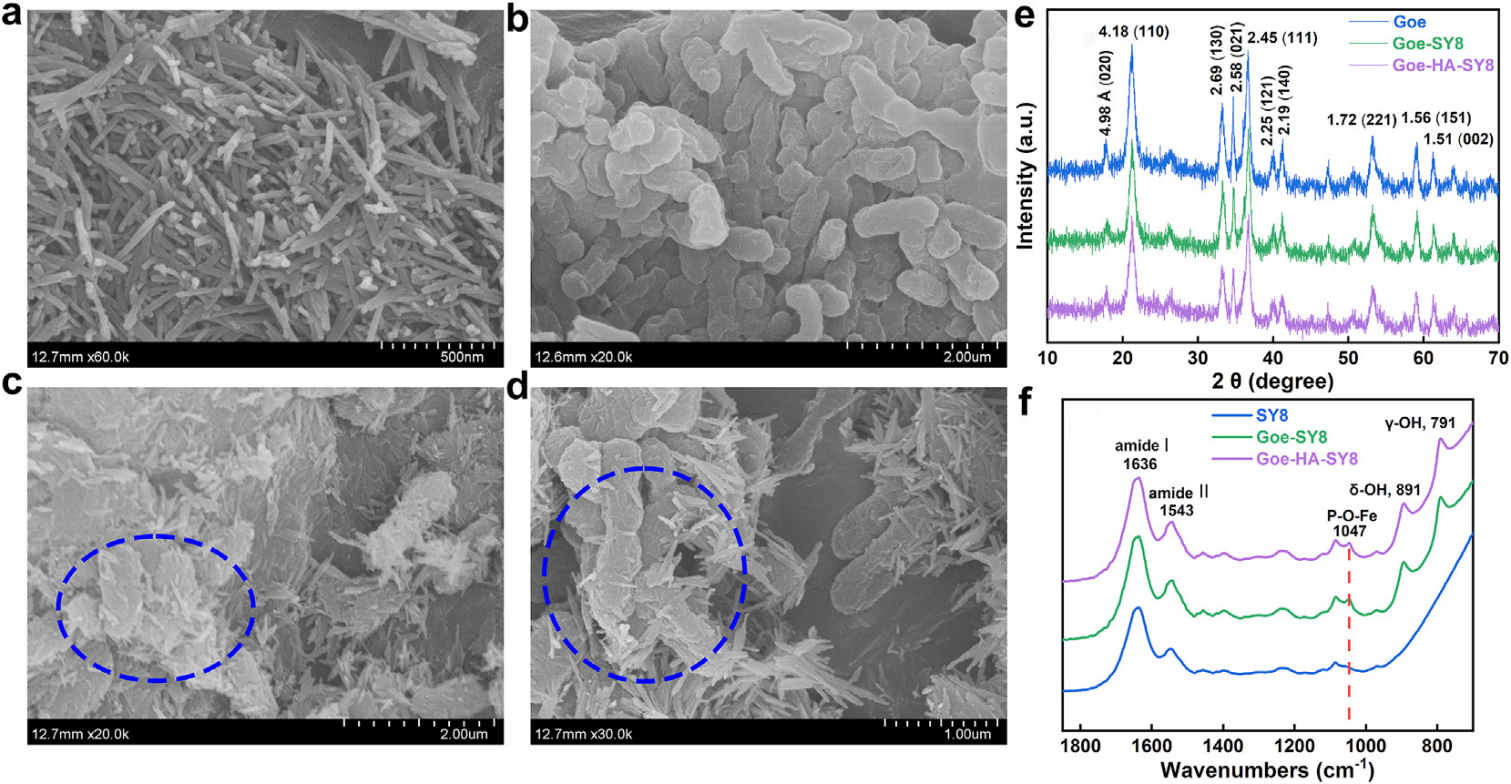
SEM images of goethite (a), SY8 (b), Goe-SY8 binary composites (c), and Goe-HA-SY8 ternary composites (d) (the dotted circles indicated the typical area of closely interaction between goethite and SY8), and XRD patterns (e) and FTIR spectra (f) of single component and their composites. Noted that the samples for XRD characterization were treated with H_2_O_2_ to exclude the interference of organic matter.

The goethite appeared as small needle-like particles with approximately 200 nm in length (Fig. 3a), while SY8 displayed an irregular rod shape with about 1 μm in length (Fig. 3b). In the Goe-SY8 composites, goethite particles were closely coated and aggregated around SY8 cells (Fig. 3c), suggesting a strong microbial-mineral interaction. The addition of HA in Goe-HA-SY8 composites slightly decreased the particle aggregation (Fig. 3d). The XRD characteristic peaks of goethite were slightly weakened after association with SY8 and HA (Fig. 3e), suggesting a decrease in crystallinity.

The FTIR spectra of the composites and band assignments are shown in Fig. 3f and Table S2. An increase in the band area for amide I at 1636 cm^−1^ and amide II at 1543 cm^−1^ was observed in both Goe-SY8 and Goe-HA-SY8 composites compared to SY8 alone (Fig. 3f), likely indicating changes in the secondary structure of proteins [44]. The emergence of a new IR vibration band at 1047 cm^−1^ in the composite spectra (Fig. 3f), indicative of P–O–Fe bond formation, suggests a biochemical interaction between the surface Fe–OH groups of goethite and the phosphate groups in the extracellular polymeric substances (EPS) and cell membranes of SY8 [45,46]. Compared to Goe-SY8 composites, the IR bands of Goe-HA-SY8 composites showed minimal changes (Fig. 3f), probably due to the low loading of HA and the potential masking of HA bands by SY8. Across a pH range of 4–10 in sterile DI water, the ζ of the components generally followed the order of goethite > Goe-SY8 > Goe-HA-SY8 > HA-SY8 ≈ HA ≈ SY8 at a given pH (Fig. S4).

**Fig. 4:**
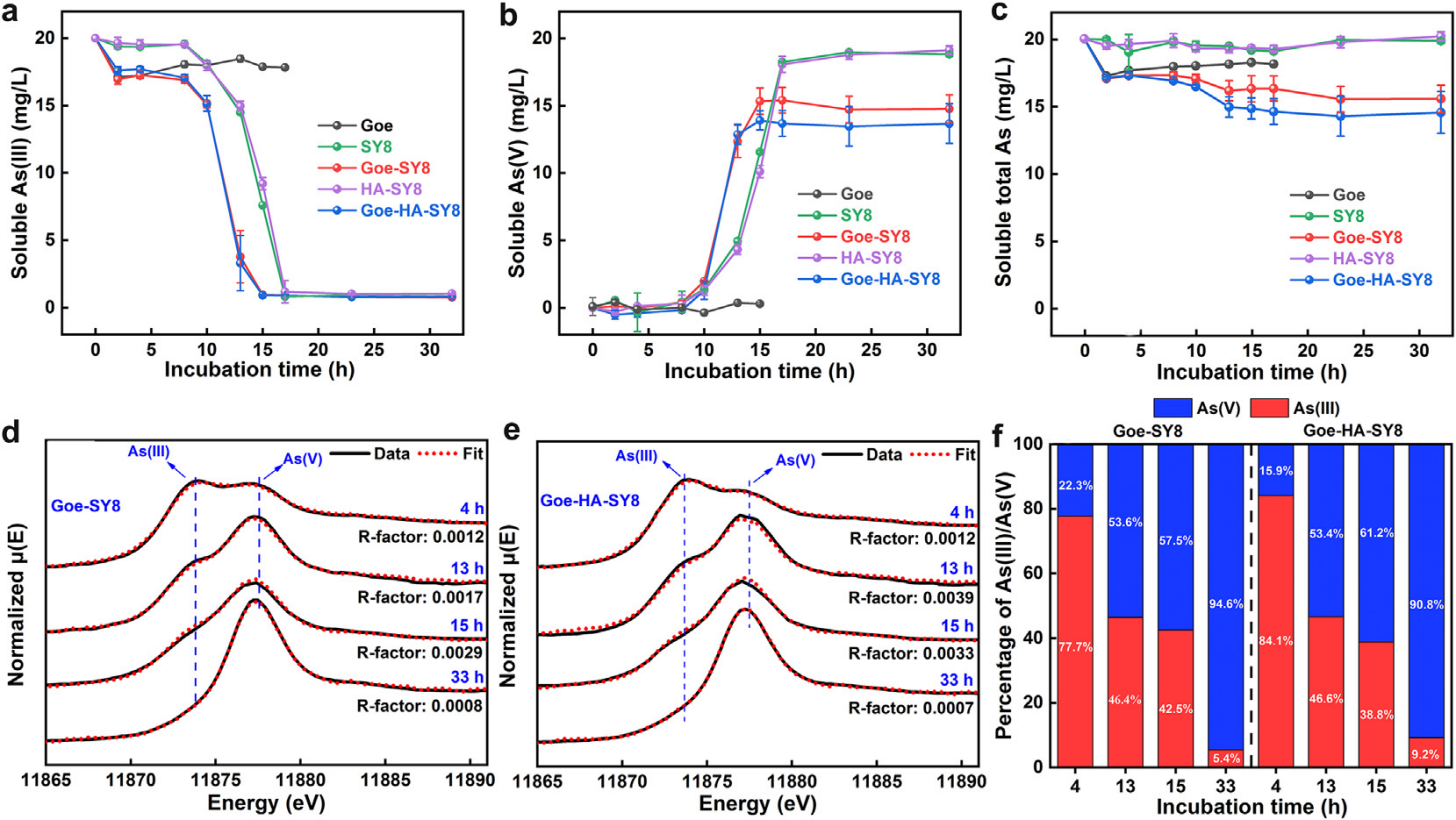
The soluble As(III) (a), As(V) (b), and total As (c) with incubation time of 33 h when SY8, goethite, HA-SY8, Goe-SY8, Goe-HA-SY8 reacted with 20 mg/L As(III) at initial pH 7, As K-edge XANES spectra and the LCF analyses of Goe-SY8 (d) and Goe-HA-SY8 composites (e) reacting with As(III) at different incubation time, and the proportions of As(III) and As(V) in the solid products (f).

The kinetics of As(III) adsorption and oxidation on single components and composites are displayed in Fig. 4, with Table S3 providing the relative proportions of soluble As(III) and As(V). These kinetics highlight the distinct roles and interactions of goethite, HA, and SY8 in As transformation processes. Under the experimental conditions, As(III) adsorption by goethite alone was relatively lower (i.e., around 3 mg/L) (Fig. 4c), compared to that of a previous report [47], likely due to competitive interactions with background ions and organic matter from the culture medium. In contrast, SY8 exhibited strong oxidative capability, converting As(III) to As(V) completely within roughly 20 h (Fig. 4a and b). This efficient transformation, combined with stable total soluble As levels (Fig. 4c), underscores SY8's potential as a key agent in bioremediation strategies, facilitating the detoxification of As through conversion to its less toxic form. In the HA-SY8 binary composites, As(III) oxidation kinetics closely resembled those observed with SY8 alone (Fig. 4a and b), indicating that HA does not notably influence the bacterium's oxidation capability. This aligns with HA's minimal effect on SY8 growth (Fig. 1c), suggesting that HA primarily affects the composite's physicochemical properties rather than its biochemical oxidation pathways.

The association of goethite with SY8 in Goe-SY8 binary composites significantly enhanced As(III) oxidation, particularly between 10 h and 15 h of incubation (Fig. 4a and b and Table S3). This observation suggests a synergistic interaction that markedly improves the oxidative transformation of As(III), highlighting the potential of leveraging mineral–microbe interactions for As bioremediation. Furthermore, Goe-SY8 composites exhibited an increased As adsorption capacity, adsorbing approximately 5 mg/L of As during incubation (Fig. 4c). This enhanced adsorption capacity underscores the role of goethite in facilitating As immobilization in tandem with oxidation.

The As K-edge XANES spectroscopy indicated that both As(III) and As(V) were adsorbed on the surfaces of Goe-SY8 composites, with the proportion of As(V) on the solids, as determined by the LCF analyses, gradually increasing from 22.3% at 4 h to 94.6% at 33 h (Fig. 4d–f). At 13 h and 15 h, the proportions of soluble As(V) were 76.6% and 94.2%, respectively (Table S3), which exceeded the corresponding proportions of As(V) adsorbed on the solids (i.e., 53.6% at 13 h and 57.5% at 15 h) (Fig. 4f). By 17 h, all soluble As had been converted to As(V) (Fig. 4b), while adsorbed As became predominantly As(V) by 33 h (Fig. 4d–f). These findings suggest that As(III) bound to goethite surfaces is more resistant to oxidation by SY8 than soluble As(III) [13,25]. This highlights the importance of optimizing conditions to enhance the bioavailability of As(III) for microbial oxidation, thereby maximizing remediation potential.

The oxidation ratios of soluble As(III) in both Goe-SY8 and Goe-HA-SY8 systems followed similar patterns (Fig. 4a), highlighting the stability of SY8's oxidative capacity in the presence of goethite, regardless of HA inclusion. In the later stages of the oxidation process (17–32 h), the Goe-HA-SY8 system exhibited a lower concentration of soluble As compared to the Goe-SY8 system by approximately 1.5 mg/L (Fig. 4b and c). This discrepancy suggests an enhanced adsorption capacity of Goe-HA-SY8 composites, potentially due to their reduced aggregation and crystallinity (Fig. 3c–e), which may increase the availability of adsorption sites for As [48]. In addition, the proportions of As(III) and As(V) in both solution and on solids were similar between the Goe-HA-SY8 and Goe-SY8 systems (Fig. 4d and Table S3), further confirming the negligible effects of HA on As(III) oxidation. Although HA does not markedly influence the oxidation efficiency of SY8 (Fig. 4a and b), it modifies the physicochemical properties of the composites (Fig. 3c–e), slightly enhancing As(V) adsorption (Fig. 4b and c). This underscores the complex role of HA in influencing the adsorptive properties of the composites without affecting the oxidative activity of SY8.

Throughout the As(III) adsorption-oxidation process, a gradual pH increase from 7.5 to 8.5 was observed (Fig. S5), likely due to OH^−^ release associated with As adsorption and H^+^ consumption during its oxidation [49]. This pH shift can significantly affect As speciation and mobility in natural environments, emphasizing the importance of pH management in As remediation. These insights into the interplay among microbial oxidation, mineral interactions, and organic matter provide a nuanced understanding of As mobility and speciation in contaminated environments. Such knowledge can inform the development of condition-specific bioremediation strategies that leverage the synergistic effects of microbial activity and mineral properties.

### 3.4 Structure and speciation variations of the composites after incubation

The XRD characteristic peaks of goethite slightly weakened with incubation time (Fig. S6a and b), likely due to the accumulation of amorphous Fe species resulting from interactions with SY8. FTIR spectral analysis of the composites revealed P-related vibration bands at 1238 cm^−1^ (PO_2_ stretching), 1120 cm^−1^ (P=O), 1085 cm^−1^ (PO_2_ stretching), and 1047 cm^−1^ (P–OFe) [44], indicative of direct interactions between SY8 and goethite through Fe–O–P inner–sphere complexes (Fig. S6c and d). The δ(OH) and γ(OH) vibration bands of goethite, observed at 891 cm^−1^ and 791 cm^−1^, respectively, also weakened with incubation time (Fig. S6c and d), mirroring trends seen in the XRD patterns (Fig. S6a and b). This suggests a modification of goethite's OH groups, potentially affecting its interaction with As and highlighting the dynamic nature of surface chemistry in these composites.

Semi-quantitative analysis of XPS full spectra indicated a decreasing trend in the Fe/C ratio over time (Fig. S7), suggestive of an increasing adsorption of carbon-bearing substances, likely EPS, on the goethite surfaces. This evolving surface chemistry confirms the microbial influence on the composite matrix, elucidating a dynamic modification that may play a critical role in As interaction dynamics.

**Fig. 5:**
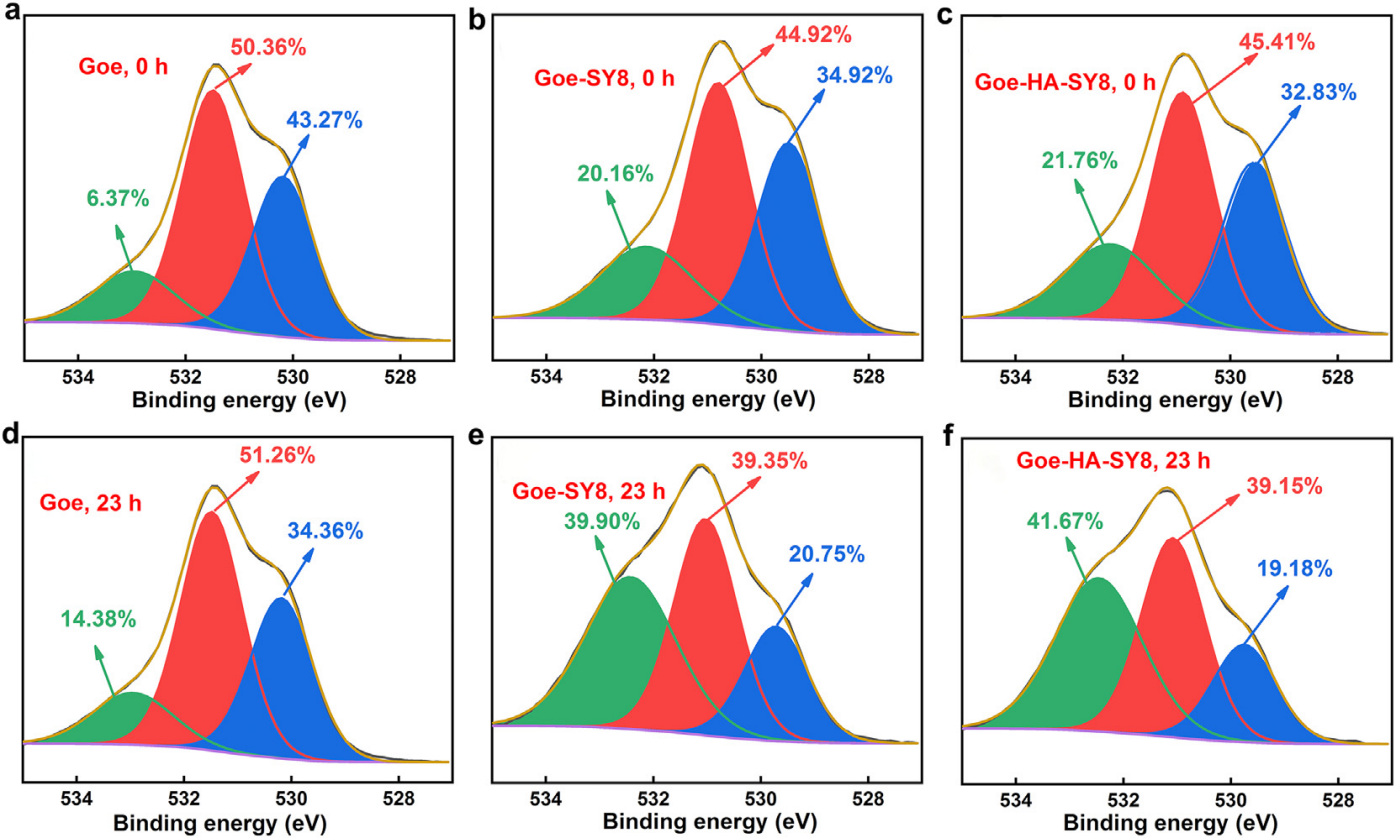
The O 1s spectra of goethite, Goe-SY8 and Goe-HA-SY8 composites before (a, b, c) and after (d, e, f) reaction with As(III).

The O 1s spectra identified three distinct O species within the composites: lattice O in Fe–O–Fe linkages at approximately 529.8 eV [27,50], surface-bound O in Fe–OH/Fe–O–As and C=O groups around 531.2 eV [51,52], and O in organic substrates and water at roughly 532.7 eV [51]. Compared to pure goethite, both Goe-SY8 and Goe-HA-SY8 composites showed a notably increased proportion of organic-related O species (around 532.7 eV), indicative of the enrichment of aldehydes, hemiacetals, and other organic groups. This trend, observed both before and after the As(III) reaction (Fig. 5), suggests an intricate chemical and/or biochemical interplay between the various components, potentially facilitating As adsorption.

After 23 h of interaction with As(III), the lattice O content (approximately 529.8 eV) in the composites decreased (Fig. 5b, c, e and f), corresponding with a reduction in goethite crystallinity (Fig. S6a and b). This decline is likely attributed to increased microbial biomass and EPS secretion over time, leading to a relative decrease in goethite within the composite matrix. Moreover, the fraction of O species at 531.2 eV diminished after 23 h of reaction, reflecting a decrease in surface OH groups on goethite, likely due to the increasing microbial biomass (Fig. S6c and d). Despite the formation of some Fe–O–As surface complexes, this observation underscores the dynamic nature of surface chemistry in response to microbial growth and As exposure, highlighting the critical role of microbial activity in modulating goethite's interaction with As.

**Fig. 6:**
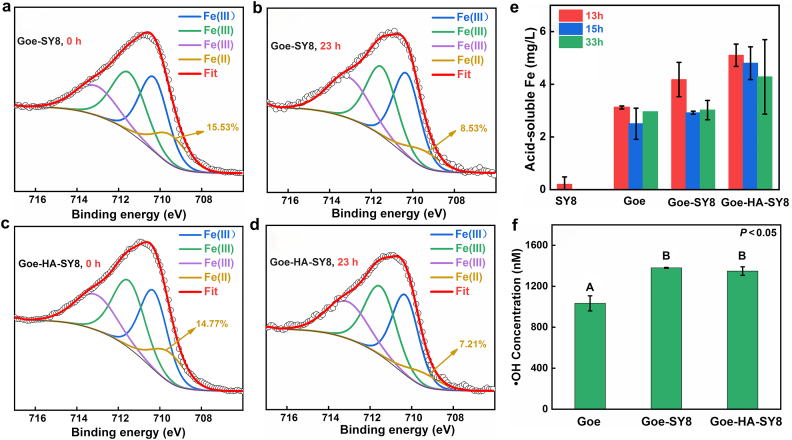
The Fe 2p_3/2_ spectra of Goe-SY8 and Goe-HA-SY8 composites before (a and c) and after reacting with As(III) for 23 h (b and d), acid-soluble Fe at different incubation time (e), and ·OH production after reacting with As(III) for 33 h (capital letters indicate significant difference) (f).

The Fe 2p_3/2_ spectral analyses (Fig. 6a–d) revealed Fe(II) species at 709.5 eV within the Goe-SY8 and Goe-HA-SY8 composites [53], indicating the formation of Fe(II) during interactions between goethite and SY8. The observed reduction in Fe(II) proportion following the As(III) reaction (Fig. 6a–d) underscores its role in the oxidative conversion of As(III), reflecting dynamic changes in Fe speciation that are critical to As remediation mechanisms. Acid-soluble Fe concentrations were notably higher in the Goe-SY8 (2.92–4.18 mg/L) and Goe-HA-SY8 (4.28–5.10 mg/L) composites compared to pure goethite (2.50–3.12 mg/L) after As(III) exposure (Fig. 6e). This suggests that Goe-SY8 interactions induce Fe speciation changes that enhance Fe dissolution. Given the negligible dissolution of goethite under weakly alkaline conditions (Fig. S5), the increased Fe dissolution is likely attributed to the formation of poorly crystalline Fe species and amorphous Fe–C complexes, as supported by XRD, FTIR, and XPS analyses (Figs. S6 and S7).

After 33 h of exposure to As(III), ·OH was detected in these systems (Fig. 6f). SY8 significantly enhanced ·OH production (Fig. 6f), emphasizing the bacterium's role in facilitating the generation of reactive oxygen species (ROS), which might be critical for the oxidative breakdown of As and other organic contaminants. These findings shed light on the intricate interactions among microbial activity, mineralogy, and As chemistry in bioremediation. The alterations in Fe speciation and the enhanced generation of ROS underscore the potential of microbial-mineral composites to optimize As remediation strategies.

### 3.5 Inhibition effects of goethite on SY8 growth

The inhibitory effects of goethite on SY8 growth can be attributed to several factors. First, goethite promotes the aggregation of SY8 due to strong electrostatic interaction between their oppositely charged surfaces (Fig. S4). This aggregation may lead to an underestimation of OD_600_ measurements. In addition, interactions between goethite and SY8 enhance the generation of ROS such as ·OH (Fig. 6f), which can damage cellular components, including lipids, proteins, and DNA, ultimately reducing cell viability and growth [54,55]. Furthermore, the close coating of goethite on the surfaces of SY8 restricts contact with substrates [56], hindering the absorption of nutrients and potentially causing structural damage to SY8. Collectively, these effects likely contribute significantly to the inhibitory impact of goethite on SY8 within the co-culture system.

### 3.6 Promotion mechanisms of goethite on As(III) oxidation by SY8

In the Goe-SY8 and Goe-HA-SY8 composites, goethite particles closely adhere to the surfaces of SY8 cells (Fig. 3c and d), which may hinder nutrient uptake by SY8 [57] and compromise cellular integrity [58]. Despite these potential inhibitory effects on SY8 growth (Fig. 1d), goethite notably catalyzes the oxidative conversion of As(III) to As(V), particularly during the incubation period of 8–17 h (Fig. 4a and b). Although initial interactions with goethite may challenge bacterial vitality, our findings indicate that goethite plays a catalytic role in enhancing As(III) oxidation. A critical factor in this enhanced As(III) oxidation involves the generation of ·OH. Our analysis revealed the presence of ·OH across the goethite, Goe-SY8, and Goe-HA-SY8 systems, with increased generation observed in systems containing SY8 (Fig. 6f). This suggests that the synergistic interaction between goethite and SY8 promotes ·OH formation, significantly enhancing As(III) oxidation at the interface of the composites.

**Fig. 7:**
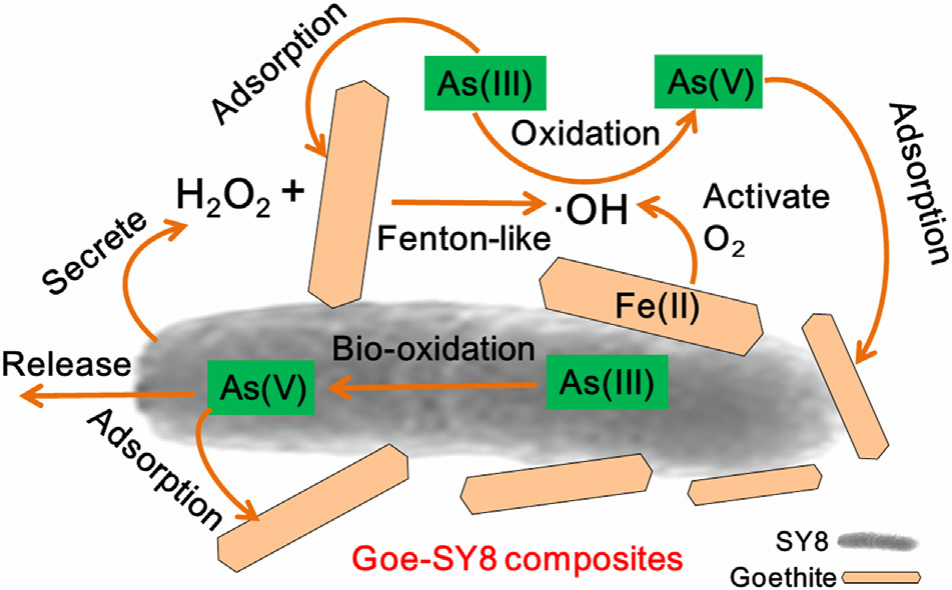
Promotion mechanisms of goethite on As(III) oxidation by SY8 during the interaction between goethite and SY8.

The generation of ·OH within the Goe-SY8 and Goe-HA-SY8 composites likely occurs through Fenton-like reactions involving two processes (Fig. 7). First, aerobic respiration by the heterotrophic microorganism SY8 produces H_2_O_2_ [59,60], which then reacts with goethite to generate ·OH through reactions such as ≡ Fe^3+^ – OH + H_2_O_2_ → ≡ Fe^2+^ + H_2_O + ·HO_2_ and ≡ Fe^2+^ + H_2_O_2_ → ≡ Fe^3+^ – OH + ·HO [39]. Additionally, the presence of Fe(II) in these composites (Fig. 6a–d) catalyzes the activation of molecular O_2_, further contributing to ·OH production (Fig. 7) [61]. These processes highlight the synergistic interaction between microbial respiration and mineral catalysis, leading to enhanced As(III) oxidation.

The role of HA in As oxidation was less pronounced, probably due to two primary factors. First, the amount of HA associated with goethite and SY8 might be low, with most remaining in solution due to repulsive forces and steric hindrance during the preparation of the composites. Second, the organic matter in the LB medium probably serves a similar function to HA, such as providing a carbon source and acting as an electron shuttle during As(III) oxidation, thus masking the effects of HA. Consequently, HA exerted minimal influence on As(III) oxidation through mineral–microbe interactions.

We propose that these findings might be applicable to a broad range of As(III)-oxidizing bacteria based on two considerations. First, many As(III)-oxidizing bacteria exhibit similar metabolic pathways for As(III) oxidation, involving transport into the periplasmic space and subsequent oxidation to As(V) by As oxidase enzymes encoded by the *aioA* and *aioB* gene clusters [7,62]. Second, these bacteria, including SY8, are typically aerobic microorganisms that produce H_2_O_2_ during respiration [59,60], which facilitates Fenton-like reactions with Fe (oxyhydr)oxide. The complex interactions between microbial metabolites and mineral components, which catalyze ·OH production and drive As(III) oxidation, illustrate the potential of microbial-mineral composites for environmental cleanup. This research highlights the promise of leveraging intrinsic microbial and mineral properties to develop effective bioremediation strategies for As-contaminated environments.

## 4 Conclusions

This study systematically elucidated the impact of goethite and HA on the adsorption and oxidation of As(III) by the bacterium SY8, uncovering the underlying mechanisms. Our findings highlight the dynamic interactions within soil component systems, particularly the complex interplay between goethite and SY8. The formation of P–O–Fe bonds, resulting in a close association between goethite and SY8, alongside observed changes in goethite crystallinity and surface chemistry, underscores the intricate mineral–microbe interactions. Despite goethite's initial inhibitory effects on SY8 growth, its interaction with SY8 facilitates the production of structural Fe(II), initiating Fenton-like reactions that enhance As(III) oxidation. These interactions have significant implications for the environmental behavior of As, influencing its toxicity, mobility, and bioavailability. Understanding these dynamics is essential for effectively addressing As contamination. Although HA does not significantly alter the oxidation process of As(III) by SY8, it enhances As adsorption on mineral surfaces by decreasing particle aggregation and mineral crystallinity. By elucidating these key interaction mechanisms, our findings contribute to a fundamental understanding of As biogeochemistry and support the development of more effective remediation strategies for arsenic-contaminated environments.

## CRediT authorship contribution statement

**Jie Deng:** Writing – original draft, Methodology, Investigation, Data curation. **Shaowei Mi:** Validation, Investigation, Data curation. **Chenchen Qu:** Writing – review & editing, Methodology. **Qiaoyun Huang:** Resources, Methodology, Funding acquisition. **Xionghan Feng:** Writing – review & editing, Methodology. **Xiaoming Wang:** Writing – review & editing, Supervision, Resources, Project administration, Methodology, Funding acquisition, Formal analysis, Conceptualization.

## Declaration of competing interests

The authors declare the following financial interests/personal relationships which may be considered as potential competing interests: Xiaoming Wang reports was provided by National Key Research and Development Program. Xiaoming Wang reports was provided by National Natural Science Foundation of China. If there are other authors, they declare that they have no known competing financial interests or personal relationships that could have appeared to influence the work reported in this paper.
