# Supplementary material for: Enhanced As(III) adsorption-oxidation via synergistic interactions between bacteria and goethite
Source: Eco Environ Health. 2024 Dec 18;4(1):100131. doi: 10.1016/j.eehl.2024.12.001 (PMC11833349; doi:10.1016/j.eehl.2024.12.001)
Supplement: Multimedia component 1 [file mmc1.docx]

**Supporting Information**

**Enhanced As(III) adsorption-oxidation via synergistic interactions between bacteria and goethite**

Jie Deng^1^, Shaowei Mi^1^, Chenchen Qu, Qiaoyun Huang, Xionghan Feng, Xiaoming Wang*

Key Laboratory of Arable Land Conservation (Middle and Lower Reaches of Yangtze River), Ministry of Agriculture and Rural Affairs, State Environmental Protection Key Laboratory of Soil Health and Green Remediation, Hubei Key Laboratory of Soil Environment and Pollution Remediation, College of Resources and Environment, Huazhong Agricultural University, Wuhan 430070, China

*Corresponding author:

Xiaoming Wang, E-mail: [wangxm338@mail.hzau.edu.cn](file:///D:\\1-graduate\\文章\\STE投稿-20210119\\wangxm338@mail.hzau.edu.cn)

^1^These two authors contributed equally to this study.

This file includes 11 pages, 3 tables, and 7 figures

**Text SI-1: Characterizations of the components**

**X-ray diffraction (XRD):** The crystal structure of solid samples was analyzed using XRD (D8-ADVANCE, BRUKER) under the following conditions: Cu Kα radiation (λ = 0.15418 nm), tube voltage of 40 kV, tube current of 40 mA, and a scanning speed of 10°/min. The scanning range was set from 5° to 85°. Prior to characterization, samples were treated with H_2_O_2_ to remove organic matter from the composites. Approximately 100 mg of solid was mixed with ~5 mL of 30% H_2_O_2_ in 50 mL centrifuge tubes and left overnight at room temperature. The mixture was filtered through a 0.45 μm membrane to obtain solid samples, which were subsequently air-dried for XRD analysis.

**Scanning electron microscope (SEM):** Morphologies of SY8, goethite, and their composites were observed using field emission scanning electron microscopy (Hitachi, Japan). Wet solids of SY8, Goe-SY8, and Goe-HA-SY8 were washed twice with PBS buffer and centrifuged at 3000 rpm for 10 min. The precipitates were fixed in 2.5% glutaraldehyde for 4 hours, washed three times with PBS buffer, and subjected to ethanol gradient dehydration (30%, 50%, 70%, 85%, and 90% ethanol, respectively, followed by 100% ethanol twice for 15 min each). After each dehydration step, the samples were centrifuged at 8000 rpm for 5 min. The final samples were pre-frozen at –20°C for 12 hours and then freeze-dried for 72 hours. Before scanning, a small amount of powder was placed on the sample table and observed after gold spraying.

**Fourier transform infrared spectrum (FTIR):** The infrared spectra of goethite, SY8, HA, and their composites before and after reaction with As(III) were obtained using Fourier transform infrared spectroscopy (VERTEX70, BRUKER). Each sample was measured over the 4000–400 cm⁻¹ range with a resolution of 4 cm⁻¹ in transmission mode. Sixty-four scans were collected for each sample, and the spectral data were processed and analyzed using the OPUS program.

**Zeta potential:** Single components and composites were dispersed in sterile deionized water. The suspension pH was adjusted to 4, 5.5, 7, 8.5, and 10 using diluted HCl or NaOH solutions. Zeta potential was measured using a Zeta potential analyzer (Zetasizer Nano ZS, Malvern). Each sample was measured three times, and the average value was used as the final zeta potential.

**X-ray photoelectron spectroscopy (XPS):** Goethite, Goe-SY8, and Goe-HA-SY8 composites before and after reaction with As(III) were analyzed by XPS (Thermo Scientific K-Alpha). Powder samples were pressed into tablets and attached to a sample plate before being placed in the XPS instrument's sample chamber, maintained at a pressure of less than 2.0 × 10⁻⁷ mbar. Measurements were conducted with a spot size of 400 μm, an operating voltage of 12 kV, and a filament current of 6 mA. Full spectrum scans were taken from 1350 eV to 9.92 eV, with pass energy and step size set at 150 eV and 1 eV, respectively. For narrow spectra of Fe 2p and O 1s, pass energy and step size were 50 eV and 0.1 eV, respectively. XPS spectra were analyzed using Avantage software, with charge correction based on the C 1s peak at 284.8 eV. A Shirley-type background was subtracted prior to deconvolution and fitting.

Text SI-2: Determination of As(III) and As(V) and ·OH concentrations

**Analysis of As speciation:** Total arsenic (As) was measured using a method involving 5 wt.% thiourea and 5 wt.% ascorbic acid as reducing and masking agents under carrier conditions of 5% HCl and 2% KBH_4_. For the determination of As(III), the carrier conditions used were 1.0% HCl and 1.0% KBH_4_. The concentration of As(V) in the solution was calculated by subtracting the As(III) concentration from the total As concentration. The analytical instrument operated with a carrier gas flow rate of 400 mL/min, a shield gas flow rate of 800 mL/min, a negative high voltage of 280 V, a lamp current of 58 mA, and an atomizer height set at 12 mm.

**Determination of ·OH:** The concentration of hydroxyl radicals (·OH) generated in the system was measured using high-performance liquid chromatography (HPLC, Agilent 1260) equipped with a fluorescence detector (FLD). A reverse-phase C18 column (ZORBAX SB-Aq, 4.6 mm × 250 mm, Agilent) was employed for the analysis. The experimental procedure involved adding 10 μM terephthalic acid (TPA) to the reaction system (goethite/Goe-SY8/Goe-HA-SY8). After the reaction, a 5 mL suspension was withdrawn, centrifuged, filtered through a sterile membrane, and stored at 4 °C for subsequent measurements. Standard concentrations of 2-hydroxyterephthalic acid (HTPA) were prepared at 0, 0.01, 0.02, 0.05, 0.1, 0.2, 0.5, and 1 μM. The HPLC system was set with excitation and emission wavelengths at 311 nm and 425 nm, respectively. The mobile phase consisted of 200 mM K₂HPO₄ and 2% (w/t) KCl mixed with acetonitrile in a volume ratio of 90:10.

Table S1 Boltzmann fitting parameters of growth curves for SY8 growth in different initial pH values, As(III) concentrations, HA concentrations, and goethite concentrations.

| Environmental conditions | | *Y*_max_ (OD_600_) | *μ*_max_ (h^-1^) | *t*_max_ (h) | *λ* (h) | R^2^ |
| --- | --- | --- | --- | --- | --- | --- |
| pH values | 5.5 | 2.04 | 0.3426 | 14.33 | 11.41 | 0.99 |
|  | 7 | 2.23 | 0.3181 | 6.75 | 3.61 | 0.99 |
|  | 8.5 | 2.21 | 0.2764 | 6.75 | 3.13 | 0.99 |
|  | 10 | 1.90 | 0.2858 | 8.98 | 5.48 | 0.99 |
| As(III) concentration  (mg/L) | 0 | 2.23 | 0.3181 | 6.76 | 3.62 | 0.99 |
|  | 20 | 2.18 | 0.3013 | 6.57 | 3.25 | 0.99 |
|  | 50 | 2.03 | 0.3191 | 8.15 | 5.02 | 0.99 |
|  | 100 | 1.88 | 0.3467 | 9.80 | 6.92 | 0.99 |
|  | 200 | 1.54 | 0.3370 | 11.57 | 8.60 | 0.99 |
| HA concentration  (mg/L) | 0 | 2.23 | 0.3181 | 6.76 | 3.62 | 0.99 |
|  | 50 | 2.26 | 0.2701 | 7.01 | 3.31 | 0.99 |
|  | 100 | 2.23 | 0.2713 | 7.04 | 3.35 | 0.99 |
|  | 200 | 2.13 | 0.2757 | 6.73 | 3.10 | 0.99 |
| Goethite  concentration  (g/L) | 0 | 2.29 | 0.3055 | 7.62 | 4.35 | 0.99 |
|  | 0.25 | 2.16 | 0.2710 | 7.58 | 3.89 | 0.99 |
|  | 0.5 | 1.90 | 0.3277 | 6.88 | 3.83 | 0.99 |
|  | 1 | 1.55 | 0.3066 | 6.52 | 3.26 | 0.99 |
|  | 2 | 0.82 | 0.3467 | 7.19 | 4.31 | 0.99 |

Table S2 The assignments of main IR bands for SY8, Goe-SY8, and Goe-HA-SY8 ^1-3^

| Wavenumber (cm^-1^) | IR band assignment | Wavenumber (cm^-1^) | IR band assignment |
| --- | --- | --- | --- |
| 1636 | amide I | 1085 | *V*_s_(PO_2_^-^) |
| 1543 | amide II | 1047 | *V* (P-OFe) |
| 1457 | δ(CH_2_) | 968 | *V* (PO_2_^-^) |
| 1397 | *V*_s_(COO-) | 891 | δ (OH) of goethite |
| 1238 | *V*_as_(PO_2_^-^) | 791 | γ(OH)of goethite |
| 1120 | *V* (P=O) |  |  |

as: asymmetric stretching vibration δ: bending vibration V: symmetric stretching vibration

Table S3 The relative proportion of soluble As(III) and As(V) at different times.

| solution samples | As(III) (%) | As(V) (%) |
| --- | --- | --- |
| SY8 10h | 92.9 | 7.1 |
| SY8 13h | 74.5 | 25.5 |
| SY8 15h | 39.6 | 60.4 |
| Goe-SY8 10h | 88.4 | 11.6 |
| Goe-SY8 13h | 23.4 | 76.6 |
| Goe-SY8 15h | 5.8 | 94.2 |
| Goe-HA-SY8 10h | 92.2 | 7.8 |
| Goe-HA-SY8 13h | 22.2 | 77.8 |
| Goe-HA-SY8 15h | 6.2 | 93.8 |

***
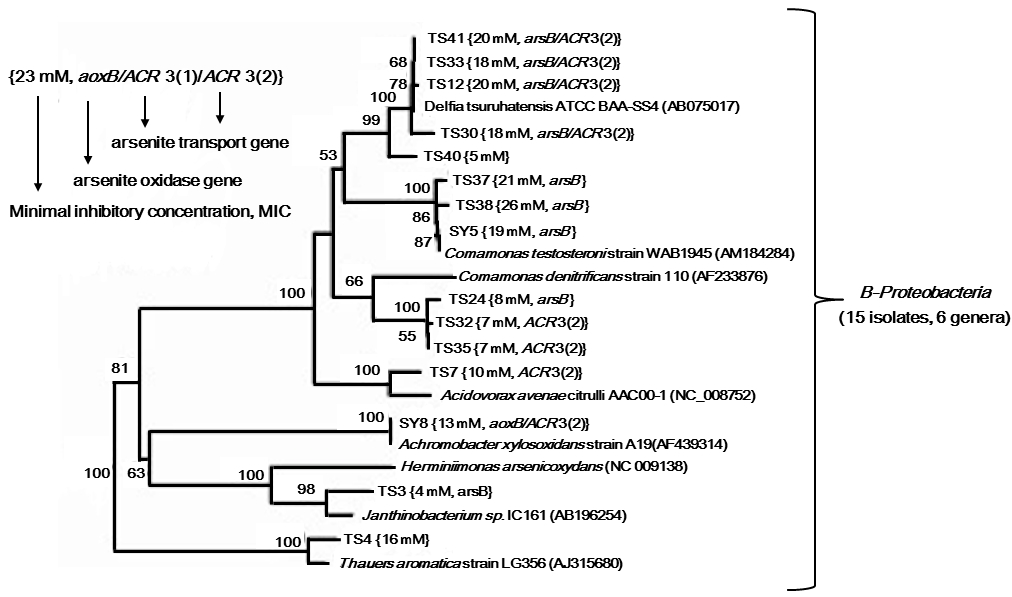
***

Fig. S1. The taxonomic information of SY8.

Fig. S2. The As(III) and As(V) references used for the LCF analyses of As K-edge XANES spectroscopy. The references were prepared by 50 mg/L As(III) or As(V) reacted with 1 g/L goethite for 4 h.

Fig. S3. The effects of initial As(III) concentration (a) and solution pH (b) on As(III) oxidation by SY8 with incubation time.

Fig. S4. The Zeta potential of different components with sterile DI water as background solution.

Fig. S5. pH changes during the As(III) adsorption-oxidation process.

Fig. S6. XRD pattern (a and b) and FTIR spectra (c and d) during of Goe-SY8 and Goe-HA-SY8 composite reaction with As(III) at different times. Note that the samples for XRD characterization were treated with H_2_O_2_ to exclude the interference of organic matter.

Fig. S7. XPS full spectra of Goe (a), Goe-SY8 composites (b), Goe-HA-SY8 composites (c) before and after reaction with As(III).

References

1. Yan, W.; Wang, H.; Jing, C. Adhesion of shewanella oneidensis MR-1 to goethite: A two-dimensional correlation spectroscopic study. *Environ. Sci. Technol.* **2016,** *50*, 4343-4349.

2. Parikh, S. J.; Chorover, J. ATR-FTIR spectroscopy reveals bond formation during bacterial adhesion to iron oxide. *Langmuir* **2006,** *22*, 8492-8500.

3. Villalobos, M.; Leckie, J. O. Surface complexation modeling and FTIR study of carbonate adsorption to goethite. *J. Colloid Interface Sci.* **2001,** *235*, 15-32.
